# A sensitivity analysis of probability maps in deep‐learning‐based anatomical segmentation

**DOI:** 10.1002/acm2.13331

**Published:** 2021-07-07

**Authors:** Noah Bice, Neil Kirby, Ruiqi Li, Dan Nguyen, Tyler Bahr, Christopher Kabat, Pamela Myers, Niko Papanikolaou, Mohamad Fakhreddine

**Affiliations:** ^1^ Department of Radiation Oncology UT Health San Antonio San Antonio TX USA; ^2^ Medical Artificial Intelligence and Automation (MAIA) Laboratory Department of Radiation Oncology UT Southwestern Medical Center Dallas TX USA

**Keywords:** deep learning, machine learning, segmentation

## Abstract

**Purpose:**

Deep‐learning‐based segmentation models implicitly learn to predict the presence of a structure based on its overall prominence in the training dataset. This phenomenon is observed and accounted for in deep‐learning applications such as natural language processing but is often neglected in segmentation literature. The purpose of this work is to demonstrate the significance of class imbalance in deep‐learning‐based segmentation and recommend tuning of the neural network optimization objective.

**Methods:**

An architecture and training procedure were chosen to represent common models in anatomical segmentation. A family of 5‐block 2D U‐Nets were independently trained to segment 10 structures from the Cancer Imaging Archive's Head‐Neck‐Radiomics‐HN1 dataset. We identify the optimal threshold for our models according to their Dice score on the validation datasets and consider perturbations about the optimum. A measure of structure prominence in segmentation datasets is defined, and its impact on the optimal threshold is analyzed. Finally, we consider the use of a 2D Dice objective in addition to binary cross entropy.

**Results:**

We observe significant decreases in perceived model performance with conventional 0.5‐thresholding. Perturbations of as little as ±0.05 about the optimum threshold induce a median reduction in Dice score of 11.8% for our models. There is statistical evidence to suggest a weak correlation between training dataset prominence and optimal threshold (Pearson r=0.92 and $p\approx 10^{‐4}$). We find that network optimization with respect to the 2D Dice score itself significantly reduces variability due to thresholding but does not unequivocally create the best segmentation models when assessed with distance‐based segmentation metrics.

**Conclusion:**

Our results suggest that those practicing deep‐learning‐based contouring should consider their postprocessing procedures as a potential avenue for improved performance. For intensity‐based postprocessing, we recommend a mixed objective function consisting of the traditional binary cross entropy along with the 2D Dice score.

## 1 INTRODUCTION

The physical process of delivering radiotherapy to cancer patients requires that some healthy tissues be irradiated. To provide an adequate treatment while minimizing the risk of toxicity, radiation treatment plans are developed based on internal anatomy visualized with computed tomographic (CT) imaging. The traditional practice of manually drawing routine sets of anatomical structures for treatment planning is not only time consuming but also subject to inconsistencies due to interobserver variability.^1^ Dice scores for the same structure drawn by different observers can commonly be in the range of 0.75 to 0.85.^2^ This has driven pattern recognition and machine learning researchers to streamline the contouring process with automatic segmentation software. Since the application of convolutional neural networks to segmentation tasks,^3^, ^4^ deep‐learning‐based automatic segmentation has become prominent in the medical physics literature.^5^ With deep learning's rapid growth and accessibility via Python libraries like PyTorch and TensorFlow, many medical physicists and radiation oncologists are entertaining ventures in automatic segmentation. The purpose of this work is to call attention to a detail of deep‐learning‐based segmentation that is often overlooked: namely, postprocessing with intensity‐based thresholding.

Deep learning models will implicitly learn to predict the presence of a structure based on its overall prominence in the training dataset. This phenomenon is observed and accounted for in deep learning applications such as natural language processing (NLP) but is often neglected in segmentation literature. In generative language modeling, the network's outputs are commonly adjusted with temperature‐based, top‐k, or more sophisticated sampling routines.^6^. This affects the so‐called “creativity” of the model on deployment and is considered mandatory for most NLP models. One major objective of these postprocessing techniques is to adjust the network output such that words that occur frequently in our language, such as “the,” are not overused.

A variety of techniques exist for postprocessing probability maps from deep‐learning‐based segmentation models; however, their importance is often understated. Some of the most thorough auto‐segmentation tutorials from Tensorflow, PyTorch, and Medium either fail to mention postprocessing at all or simply round the network output to either zero or one.^7^, ^8^, ^9^ The main contribution of this work is to empirically demonstrate that the most frequently used technique for postprocessing in deep segmentation, intensity‐based classification of pixels with a threshold of 0.5, is often insufficient for contouring anatomical structures. We will show that the perceived generalization accuracy of a segmentation model postprocessed with intensity‐based thresholding can be significantly affected for even small deviations from the true optimal threshold. Furthermore, we will provide evidence that the optimal threshold value for intensity‐based postprocessing depends significantly on the volume of the anatomical structure in the training dataset. Finally, we will recommend an amendment to the usual binary cross entropy objective to thwart thresholding sensitivity altogether. With our analyses, we demonstrate the importance of careful use of segmentation models and the potential biases and sensitivities of different metrics.

Before we continue, we would like to reiterate that the common neglect of postprocessing is not due to a lack of sufficient techniques. Some postprocessing methods that have been shown to improve model performance utilize conditional random fields and superpixels.^3^, ^10^, ^11^, ^12^ These techniques usually rely on traditional segmentation models to augment deep learning outputs. Developers seeking state‐of‐the‐art segmentation results should consider these routines as an alternative to intensity‐based postprocessing.

## 2 METHODS

Our strategy is as follows:

1. Construct a standard deep‐learning segmentation architecture and training procedure.
2. Train our standard model to segment a variety of clinically relevant structures.
3. Compare the validation accuracy of the models with varying intensity‐based postprocessing procedures.

**Figure 1:**
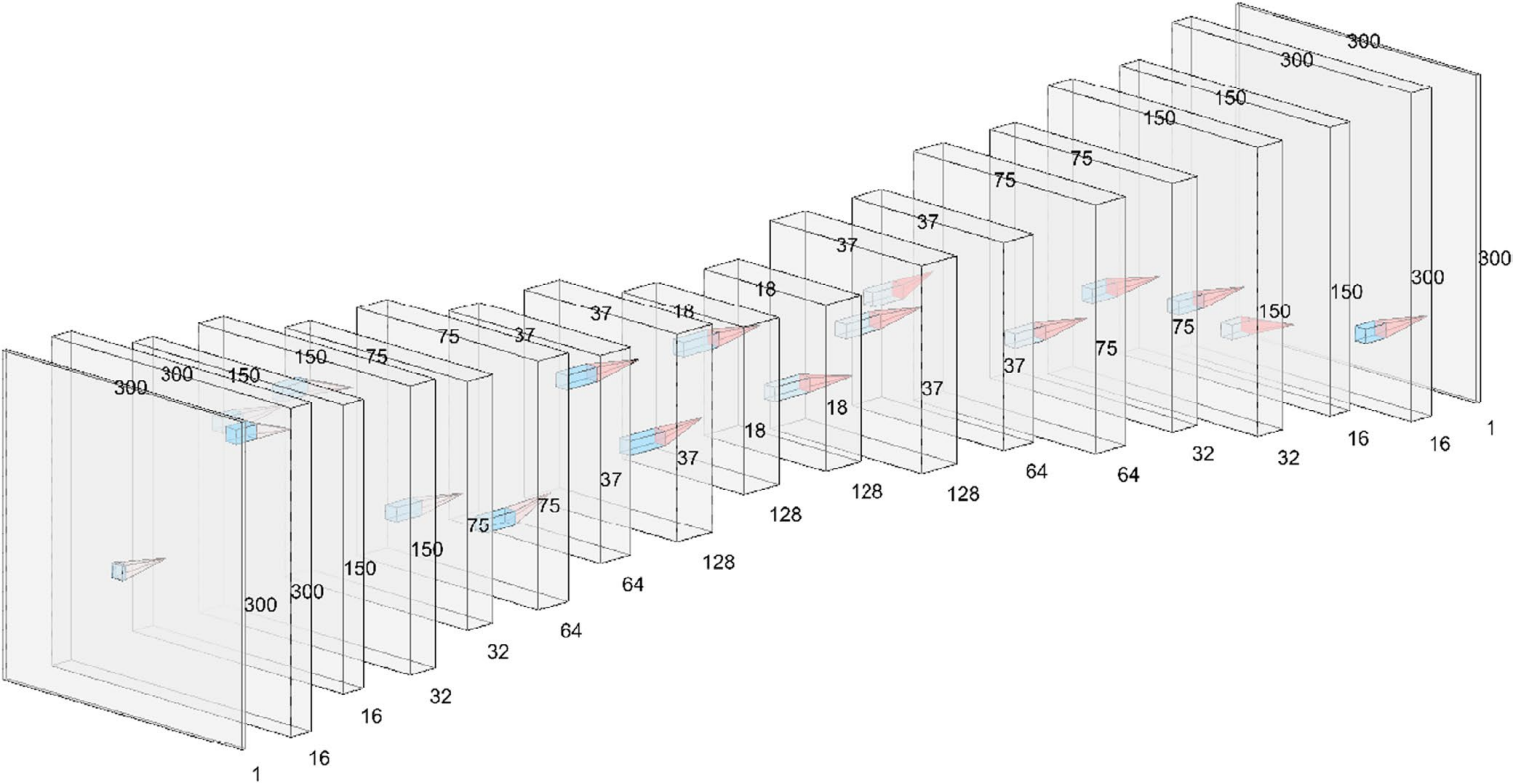
Our U‐Net architecture. Skip connections are not pictured^13^

We first construct a standard deep‐learning model used in biomedical image segmentation. The model is a 2D U‐Net with five convolutional down‐blocks and 3 × 3 kernels. The model uses five up‐blocks with strided transposed convolutions for smart up‐sampling and the usual skip connections to preserve low‐level features in the final probability map (Figure 1). During training, we use 20% dropout and batch normalization with a batch size of 64. To prevent overfitting, we conduct a form of early stopping in which we monitor the running average validation loss over the course of the last 10 epochs and store the model having the lowest error. Networks were fit using an Adam optimizer against a binary cross entropy (BCE) objective with a learning rate of 0.001. Each model was trained for a maximum of 500 epochs on an NVIDIA GeForce 2080 GPU, taking about 1.5 h per model. All models were constructed with the latest stable version of PyTorch, version 1.8.1.

**Figure 2:**
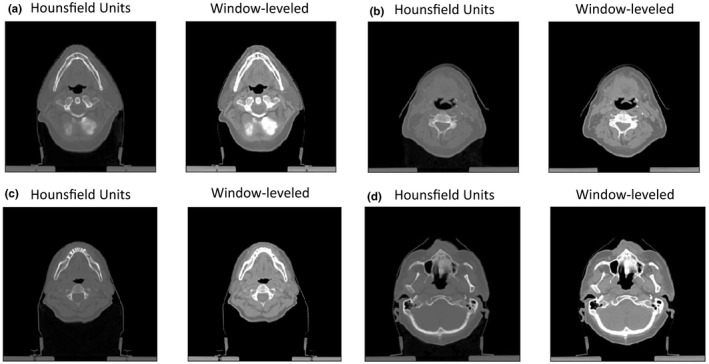
Moderate windowing and leveling improves soft tissue contrast, which is necessary for contouring structures in the head and neck. Modified images (right) following window‐leveling preprocessing are shown here for some example slices (original images on left)

Using the outlined training procedure, 10 individual models were trained to contour normal structures in the head and neck. Images and radiotherapy structures were obtained in the DICOM format from the Cancer Imaging Archive's Head‐Neck‐Radiomics‐HN1 dataset.^14^ The structure coordinates were reinterpreted as binary masks. A total of 124 patients (16 756 slices) were used for training, eight patients were used for validation (1068 slices), and five for testing (776 slices). The images were preprocessed with center cropping to 300 × 300 pixels. We found that all structures were more easily distinguishable following some mild windowing and leveling (Figure 2). Each dataset was windowed and leveled identically, normalized from 0 to 1 and stored as PyTorch floats.

**Figure 3:**
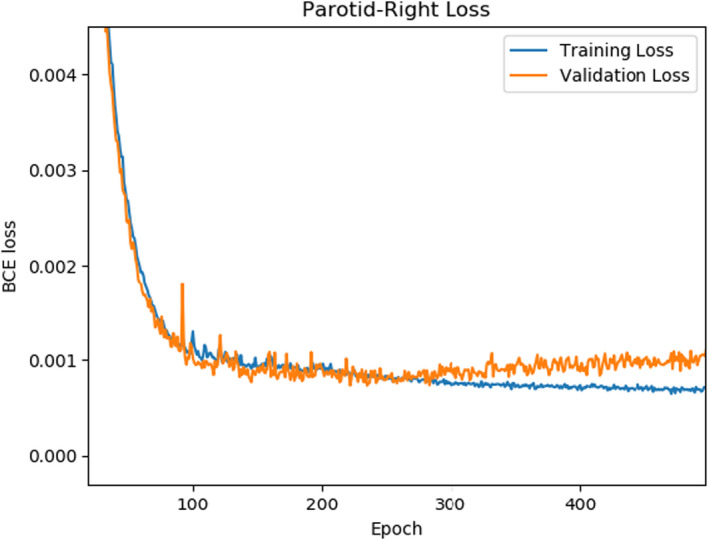
Networks were trained for 500 epochs each. A running average of the 10 most recent validation loss scores was maintained, and the model with the lowest score was stored for deployment

**Figure 4:**
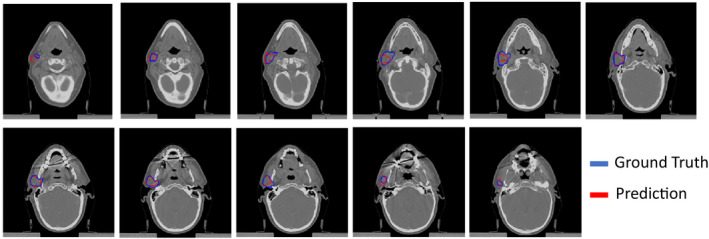
Predicted segmentations for a test patient's right parotid

**Figure 5:**
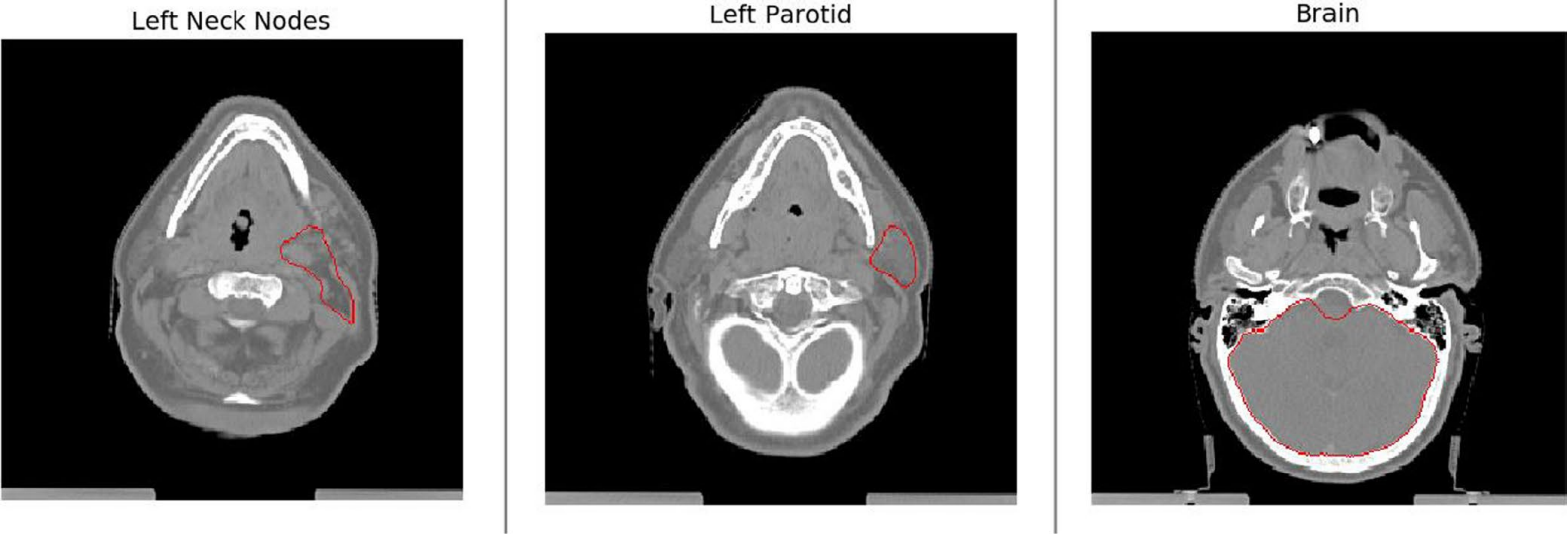
Predicted segmentation of a test patient's brain, left neck nodes, and left parotid

The images and structures were then resampled to an input size of 150 × 150 pixels. During training, we observed the expected decrease of training and validation loss for each model (Figure 3). Upon deployment, the models contour structures at about 30 slices per second. Our networks appeared to contour structures on the validation CTs within the expected accuracy range for these architectures and data (Figures 4 and 5).

The performances of the trained models were analyzed with the validation data after postprocessing with various intensity thresholds. The employed thresholding procedure can be described as follows:

Given a 2D map of sigmoid‐activated network outputs with pixels' intensities $P_{i}\in \left(0,1\right)$, let $P_{i}\to \theta \left(P_{i}‐t\right)$ for some threshold t, where θ is the Heaviside step function.

This technique is most frequently implemented by choosing t = 0.5, implying that the network is equally “confident” in declaring a structure present or not present. In our analysis, we explore our models’ accuracies as a function of threshold t. To assess the networks’ performances, we use the conventional 3D Dice coefficient.^15^ For a prediction $P^{\left(j\right)}$ and Boolean mask $M^{\left(j\right)}$, the Dice coefficient is

(1) $$D^{\left(j\right)}\left(t\right)=2*\frac{\sum_{i}\theta \left(P_{i}^{\left(j\right)}‐t\right)*M_{i}^{\left(j\right)}}{\sum_{i}\theta \left(P_{i}^{\left(j\right)}‐t\right)+M_{i}^{\left(j\right)}},$$

where indices i and (j) are meant to label voxels and training examples, respectively. The Dice coefficient is bounded between 0 and 1, with typical deep‐learning‐based segmentations scoring anywhere from 60% to 90% depending on the training procedure, architecture, and dataset. As part of our analysis, we determine the optimum threshold $t^{*}$ for each model by evaluating the validation Dice score for thresholds varying from 0 to 1 by 0.01. For validation masks ${\{M}^{\left(1\right)},M^{\left(2\right)},\cdots ,M^{\left(m\right)}\}$ and predictions ${\{P}^{\left(1\right)},P^{\left(2\right)},\cdots ,P^{\left(m\right)}\},$

(2) $$t^{*}=\underset{t\in \left(0,1\right)}{\text{argmax}}\sum_{j=1}^{m}D^{\left(j\right)}\left(t\right)$$

We then consider changes in the models’ accuracy ε under perturbations η about the optimum threshold:

(3) $$\varepsilon =\frac{1}{2}\left(\left|D\left(t^{*}+\eta \right)‐D\left(t^{*}\right)\right|+\left|D\left(t^{*}‐\eta \right)‐D\left(t^{*}\right)\right|\right)$$

**Figure 6:**
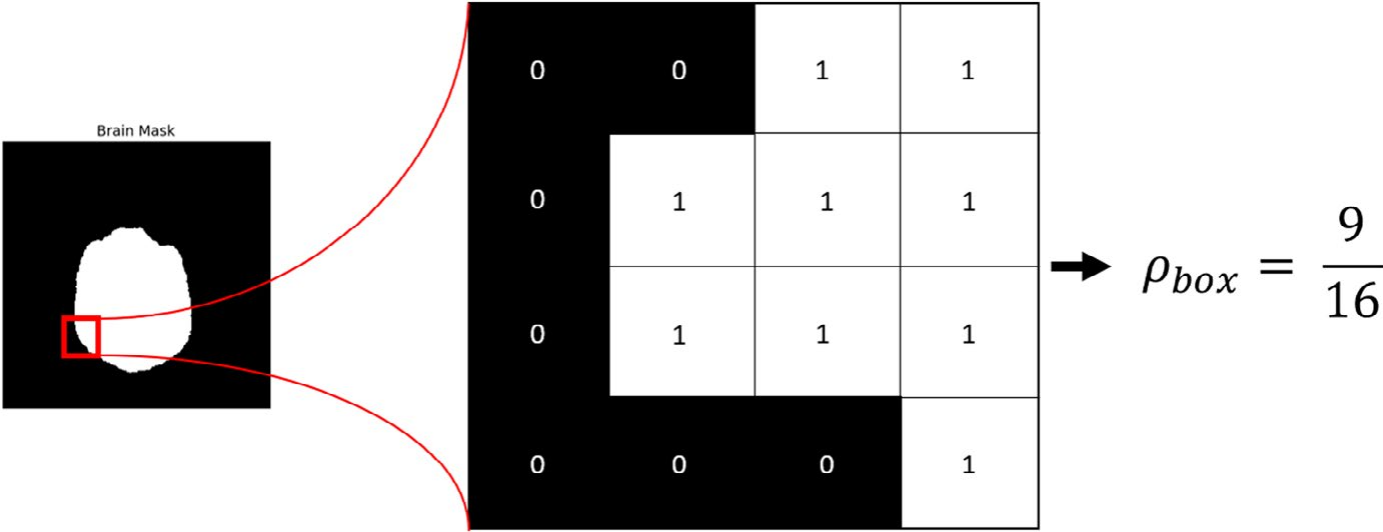
Example calculation of the fraction of “true” pixels in a region of a brain mask from the training dataset. The prominence of a training dataset is the total fraction of voxels labeled “1” in the set of Boolean masks

Next, we assess the dependence of the optimal threshold value upon the presence of each structure in the datasets. The prominence of structures was quantified by counting the fraction of total voxels in the dataset, which are labeled “true” in the Boolean masks (Figure 6). For training dataset $\left\{\left(x^{\left(1\right)},M^{\left(1\right)}\right),\left(x^{\left(2\right)},M^{\left(2\right)}\right),\cdots ,\left(x^{\left(m\right)},M^{\left(m\right)}\right)\right\}$ with CT images $x^{\left(j\right)}$ and masks $M^{\left(j\right)}$, we define a structure's prominence ρ as

(4) $$\rho =\frac{\sum_{j=1}^{m}\sum_{i}M_{i}^{\left(j\right)}}{\sum_{j=1}^{m}\sum_{i}1}$$

For each anatomical segmentation, we evaluate the structure's prominence and compare it with the threshold that yields the best average Dice score on the validation dataset.

Notice that the prominence ρ as it is defined is summed over the entire training dataset. This is specified to test the hypothesis that having a scarcity of “true” voxels in the *training* dataset will reduce the optimal threshold for that dataset at the time of deployment, or $\frac{dt^{*}}{d\rho }>0.$

Lastly, we consider stabilizing the model outputs by optimizing the network with respect to the 2D Dice score itself. This is one popular method to resolve the instabilities caused by class imbalance. However, the Dice score tends to misrepresent segmentation accuracy when structure is relatively scarce within a slice. This happens because edge pixels, which are more likely to be misclassified, contribute more significantly to the total area. This suggests that models trained with a Dice loss might unfavorably prioritize the segmentation of slices with relatively small structure areas. To demonstrate this effect, we train several models to segment the brain with objective functions of the form

(5) $$L\left(P^{\left(i\right)},\;M^{\left(i\right)}\right)=\left(1‐\chi \right)\left[‐\sum_{j}M_{j}^{\left(i\right)}\log\left(P_{j}^{\left(i\right)}\right)+\left(1‐M_{j}^{\left(i\right)}\right)\log\left(1‐P_{j}^{\left(i\right)}\right)\right]+\chi \left[1‐2\sum_{j}\frac{P_{j}^{\left(i\right)}*M_{j}^{\left(i\right)}+1}{P_{j}^{\left(i\right)}+M_{j}^{\left(i\right)}+1}\right]=\left(1‐\chi \right)\;\left[\mathrm{BCE Loss}\right]+\chi \;\left[\mathrm{Dice Loss}\right]$$

where χ is a hyperparameter between 0 and 1 determining which term if any should be favored.

The models’ performances under variable χ are assessed using the validation data with respect to the 3D Dice score (Equation 1) and mean surface distance (Equation 8). The mean surface distance is an additional metric used to quantify the accuracy of segmentation models.^16^ For a point x and surface S, we can identify the minimum Euclidean norm

(6) $$d\left(x,S\right)=\min_{x^{\prime }\in S}\left|\left|x‐x^{\prime }\right|\right|$$

which characterizes the minimum distance between the point and the surface. Then for surfaces P and M defined by the network outputs and masks, the mean surface distance (MSD) can be written

(7) $$MSD\left(P,\;M\right)=\;\frac{1}{n_{P}+n_{M}}\left[\sum_{i=1}^{n_{P}}{d(P}_{i},\;M)+\sum_{i=1}^{n_{M}}{d(M}_{i},\;P)\right]$$

where $n_{P}$ and $n_{M}$ are the number of voxels on surfaces P and M, respectively. Contrary to the Dice coefficient, lower MSD scores are indicators of good performance.

Additionally, we consider a third metric, the symmetric Hausdorff (HD) distance are shown. Symmetric Hausdorff distance is another common measure of segmentation model performance. It is defined as

(8) $$HD\left(p,\;M\right)=\max\left[\max_{x\in P}\;d\left(x,\;M\right),\;\max_{x\in M}d\left(x,\;P\right)\right]$$

The symmetric HD intuitively finds the largest distance or “worst case” voxel pair between the prediction and the target. As with MSD, lower scores indicate a better performance.

We hope to show that the stability afforded by including 2D Dice in the optimization objective comes at a cost. It is expected that despite the stabilization of the models with respect to threshold as assessed with Dice, we will find that the Dice‐optimized models can perform worse when assessed with distance‐based metrics.

## 3 RESULTS

**Figure 7:**
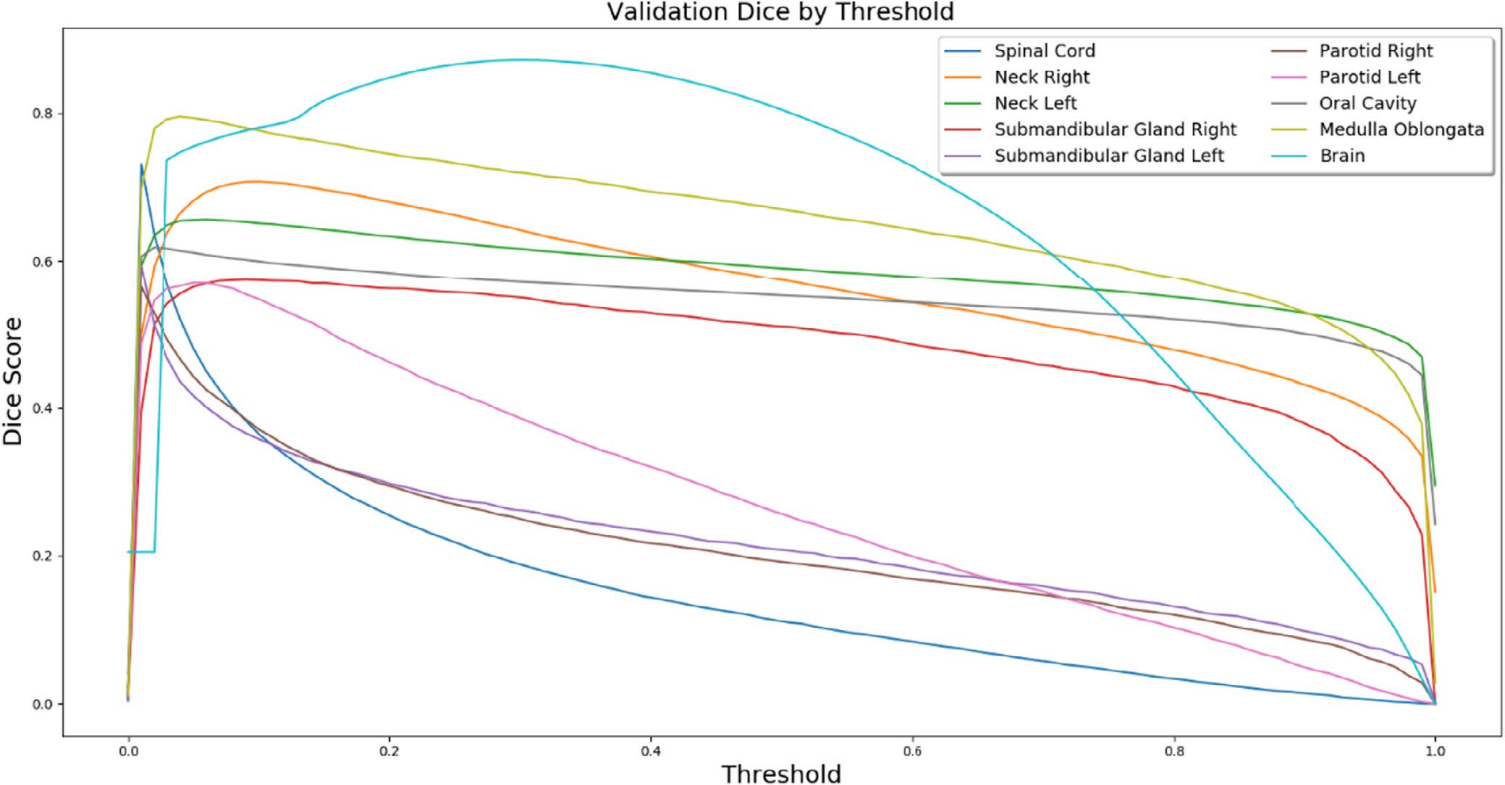
Validation Dice score depends significantly on threshold postprocessing

The 3D validation Dice scores for each model at various thresholds were determined (Figure 7). Notice that the threshold with the highest validation accuracy is not 0.5 for any of the datasets, and the optimal threshold for most models is near 0.1. Another notable attribute of this figure is that some of the curves, particularly those of small structures such as the spinal cord, are very sharply peaked. The perceived accuracy of these models, when scored with the Dice coefficient, is widely susceptible to errors in postprocessing.

**Figure 8:**
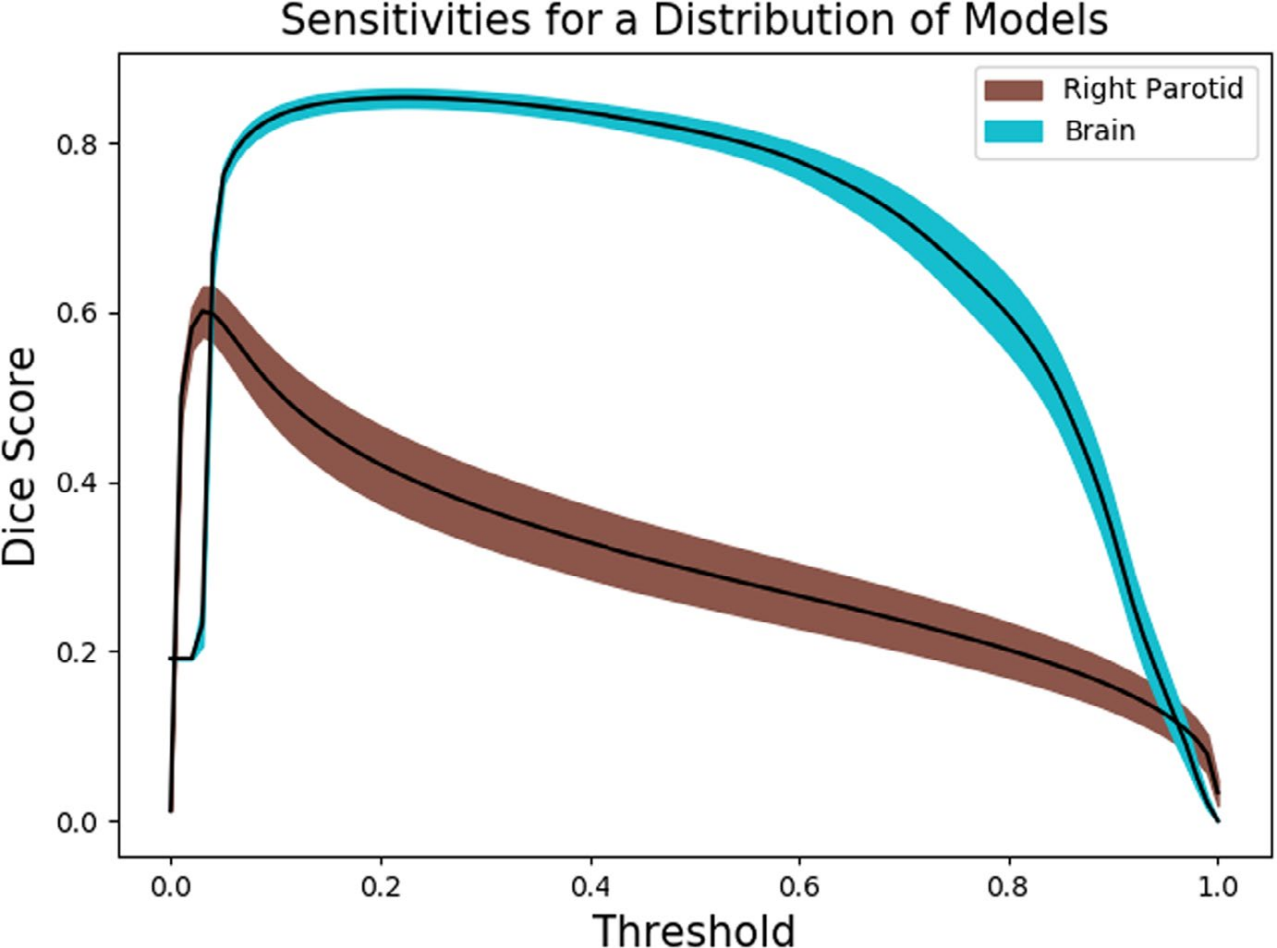
Model sensitivities to threshold postprocessing for both large and small structures are relatively stable when assessed with Monte Carlo cross‐validation

To test that these sensitivities are not anomalous, we performed Monte Carlo cross‐validation with an 80–20 train‐test split for 30 samples and show sensitivity curves with error bars for the brain and right parotid segmentation models (Figure 8). The bottom and top bounds for each curve are specified by ±1 standard error of the mean validation Dice score.

**Figure 9:**
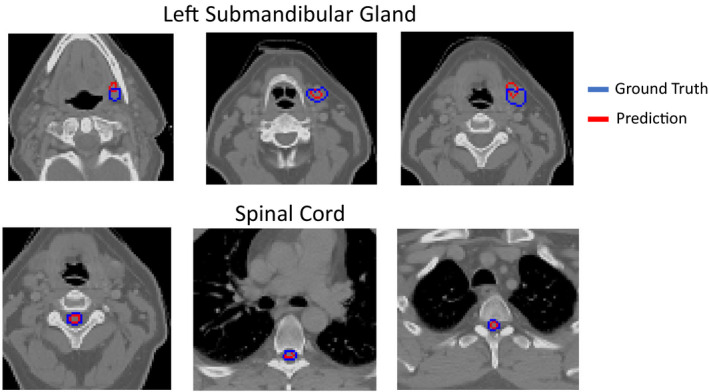
Submandibular gland and spinal cord segmentation with this training procedure achieves a validation Dice score of about 60%, despite reasonable contours

Note that a low Dice score is not an outright assertion that the segmentation is of low quality. In cases such as submandibular gland segmentation when the targets are especially small, the edge pixels contribute significantly to the intersection volume in the Dice calculation. This can somewhat misleadingly deflate scores of what might otherwise be considered clinically useful segmentations (Figure 9).

**Figure 10:**
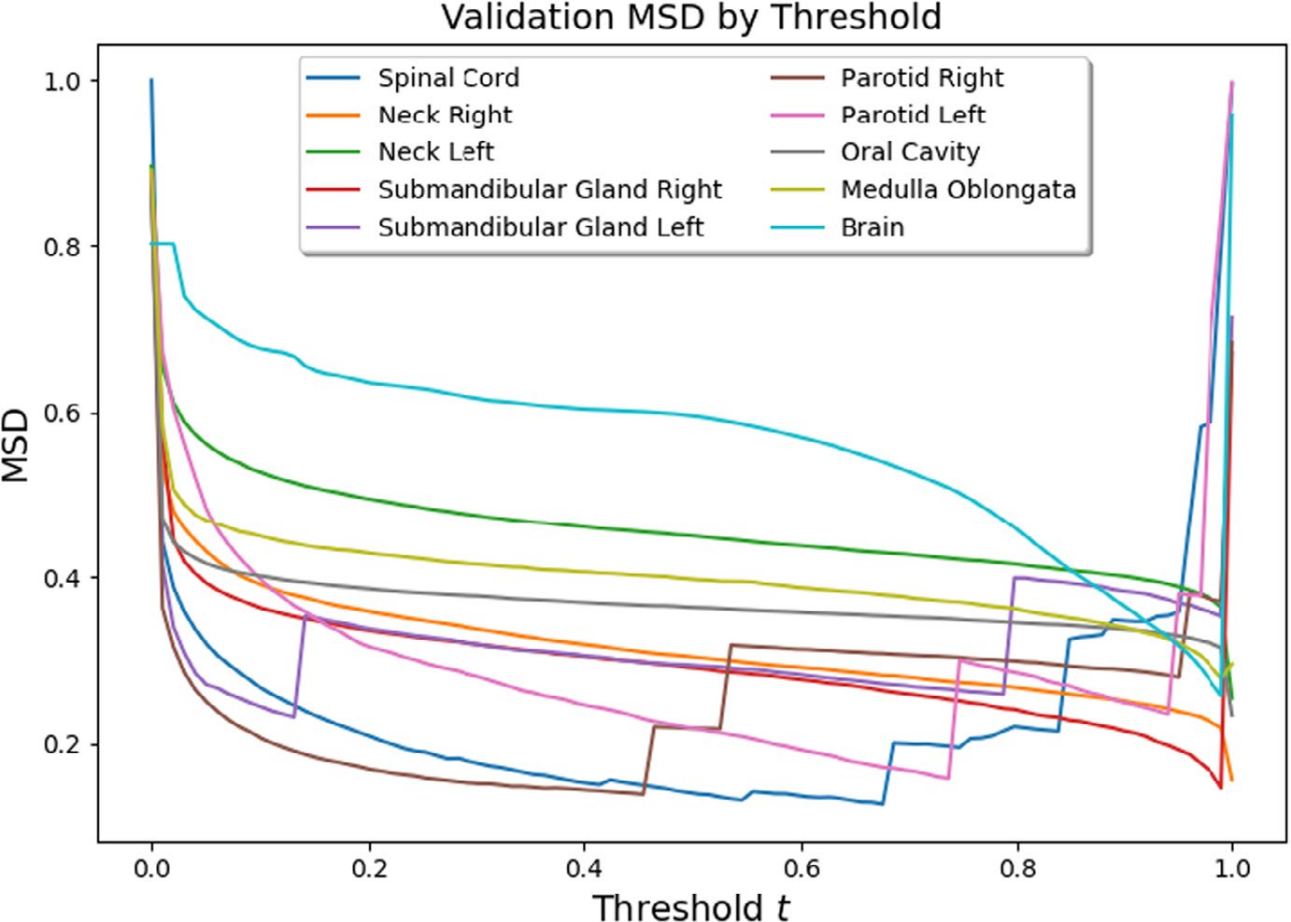
Thresholding sensitivity with respect to mean surface distance

**Figure 11:**
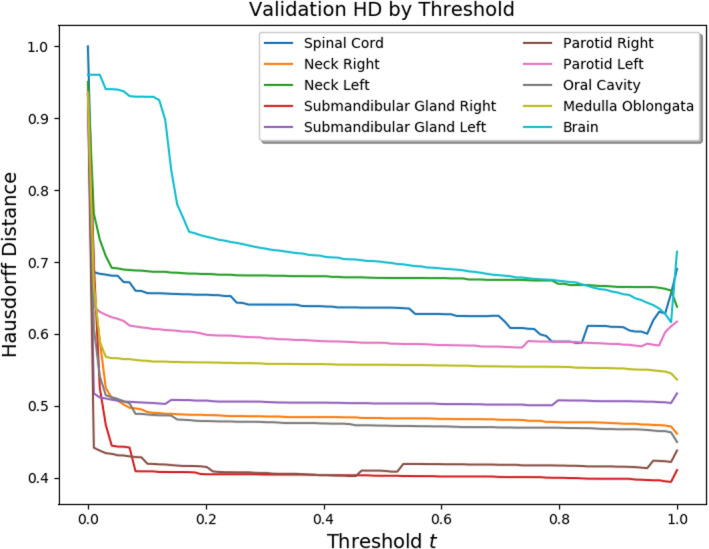
Thresholding sensitivity with respect to symmetric Hausdorff distance

We found that, when assessed with other metrics such as MSD, the performance of the submandibular gland and spinal cord models actually surpassed the brain model. As in Figure 7, one can investigate thresholding sensitivity with respect to other metrics (Figures 10 and 11).

Notice that models for large structures such as the brain which performed especially well when assessed with the 3D Dice coefficient pale in comparison with small structure models such as those for the submandibular glands and spinal cord when assessed with MSD or HD. This, as expressed above, is likely due to misclassification of edge pixels in the contouring of smaller structures. Additionally, these plots suggest that often the optimal threshold is closer to 1 than 0, contrary to the results from the 3D Dice. Upon visual inspection, we preferred thresholds recommended by the Dice analysis. This, however, suggests an imperfection in our performance measures: that an unbiased assessment of segmentation performance should consider various metrics.

**Figure 12:**
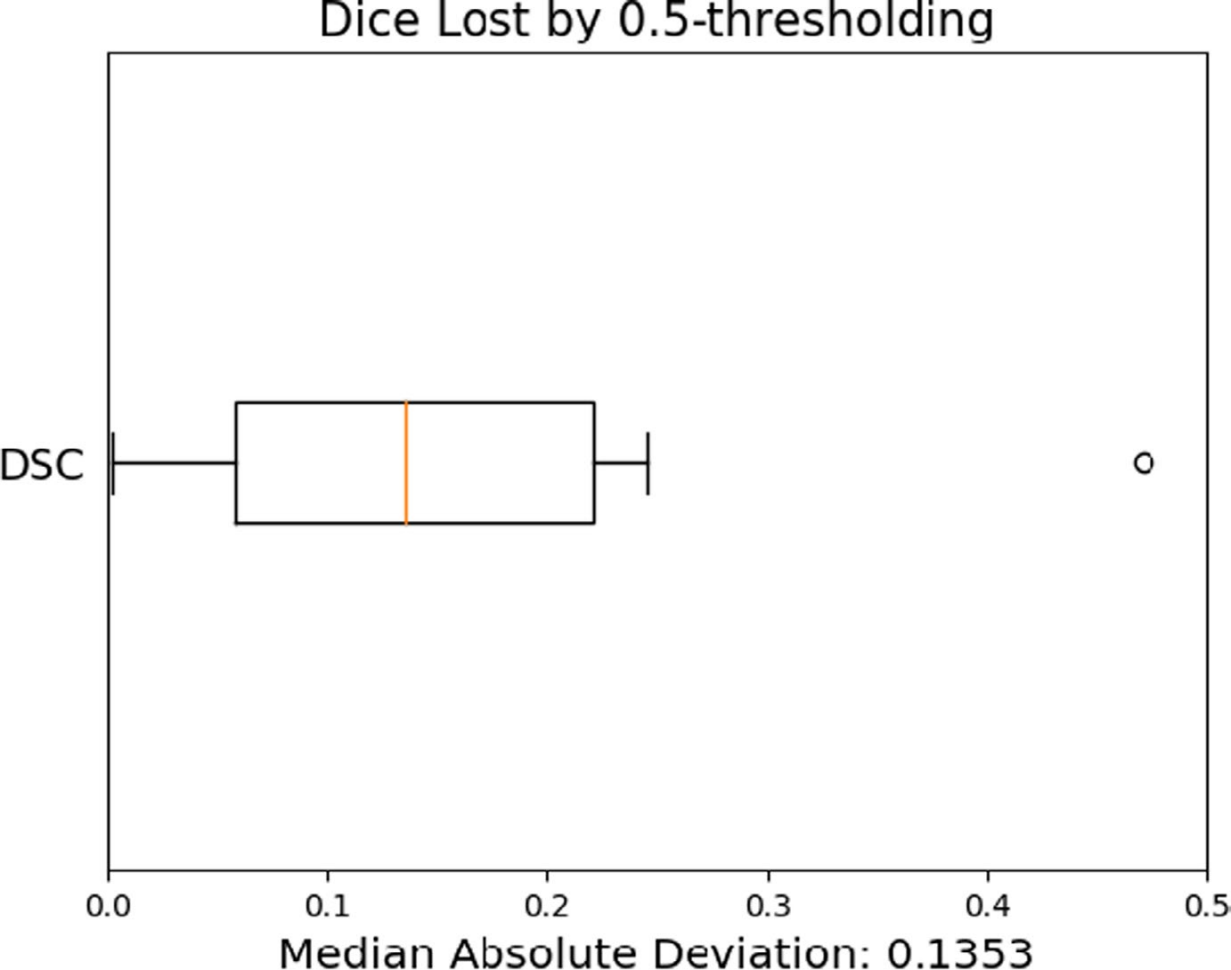
Accuracy reduction due to 0.5‐thresholding. A median Dice score deviation of 13.5% is observed

To illustrate the inadequacy of 0.5‐thresholding, we have included the distribution of Dice score losses for each of the models when compared with optimal thresholding (Figure 12). Optimal thresholds were found as outlined in Equation (2) using the validation data, and the errors $\bar{D}\left(t^{*}\right)‐\;\bar{D}\left(0.5\right)$ were evaluated using the test dataset.

The decrease in segmentation accuracy depends significantly on the structure in question. The deviation of Dice scores from their optimum value is largely driven by the inclusion or exclusion of edge pixels. However, even for large structures such as the whole brain, we expect that deviations of up to 5% are typical. We suspect that the median deviation of 13.5% is an overestimate for general anatomical segmentation errors, because structures in the head and neck are atypically small.

**Figure 13:**
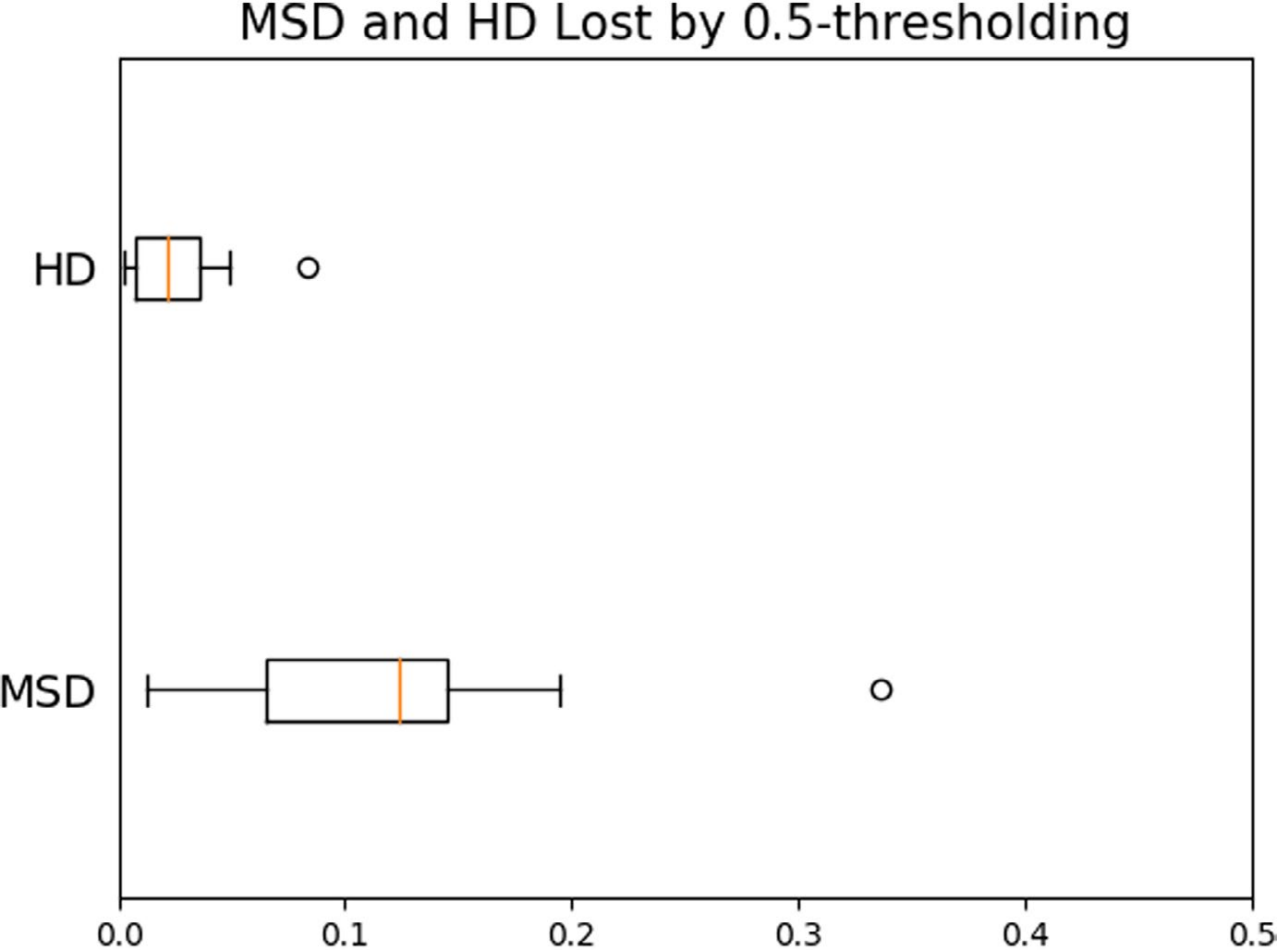
Accuracy reduction due to 0.5‐thresholding as assessed with metrics other than the Dice score. Median decreases of 12.3% and 2.1% were observed for MSD and HD, respectively

Similarly, we can evaluate optimal thresholds according to other metrics such as MSD and HD and evaluate perceived accuracy losses when assessed with these metrics. Distributions of $\left|MSD\left(t_{\mathrm{MSD}}^{*}\right)‐MSD\left(0.5\right)\right|$ and $\left|HD\left(t_{\mathrm{HD}}^{*}\right)‐HD\left(0.5\right)\right|$ for the head and neck structures are shown in Figure 13. While MSD is sensitive to the same degree as the Dice score, having median absolute decrease in performance by 12.3%, the symmetric HD appears to be relatively robust to threshold postprocessing, with a median decrease of only 2.1%.

**Figure 14:**
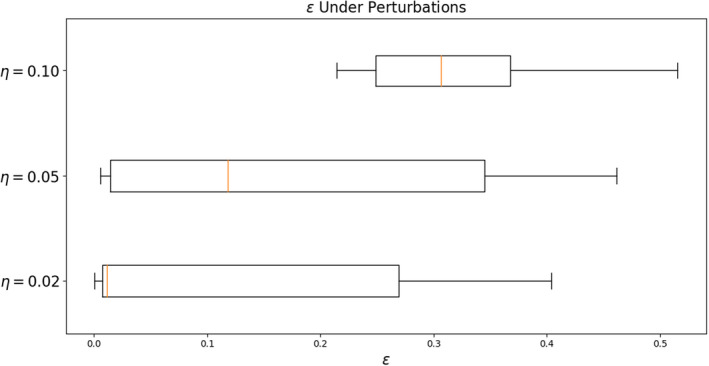
Changes to the optimum threshold of only 0.05 yield a median Dice score loss of 11.8%. High‐resolution threshold sampling is essential for intensity‐based thresholding of U‐Net outputs

Errors in segmentation accuracy were assessed under perturbations to the optimal threshold as in Equation (3). We found in most cases that the Dice score decrease caused by using a threshold as little as ±0.02 from the optimum was significant (Figure 14). With perturbations about the optimum threshold (η)=0.02,0.05, and 0.10, we find median errors ϵ=1.2%,11.8%, and 30.6%, respectively. For optimal intensity‐based thresholding, especially in the head and neck, we recommend a high‐resolution sampling of thresholds on a validation dataset.

**Figure 15:**
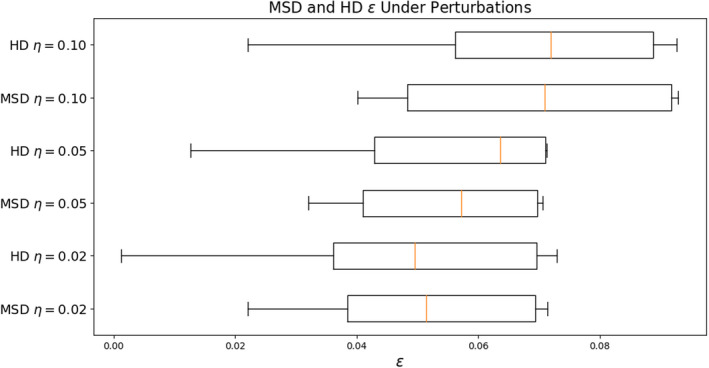
MSD and HD are relatively stable under perturbations about the optimal thresholds $t_{\mathrm{MSD}}^{*}$ and $t_{\mathrm{HD}}^{*}$ compared with 3D Dice. For perturbations about the optima of η=0.05, we find median ϵ of about 6% for both measures

An identical analysis for MSD and HD metrics shows that perturbations of (η)=0.02,0.05, and 0.10 have a less significant (but still quite large) effect on model performance as assessed with these metrics (Figure 15). For MSD, we find median decreases in performance of 5.1%, 5.7%, and 7.1% for the respective perturbations. For HD, we observe decreases of 5.0%, 6.4%, and 7.2%. Again, we expect that these metrics are less sensitive to the inclusion or exclusion of boundary pixels than the Dice score.

**Figure 16:**
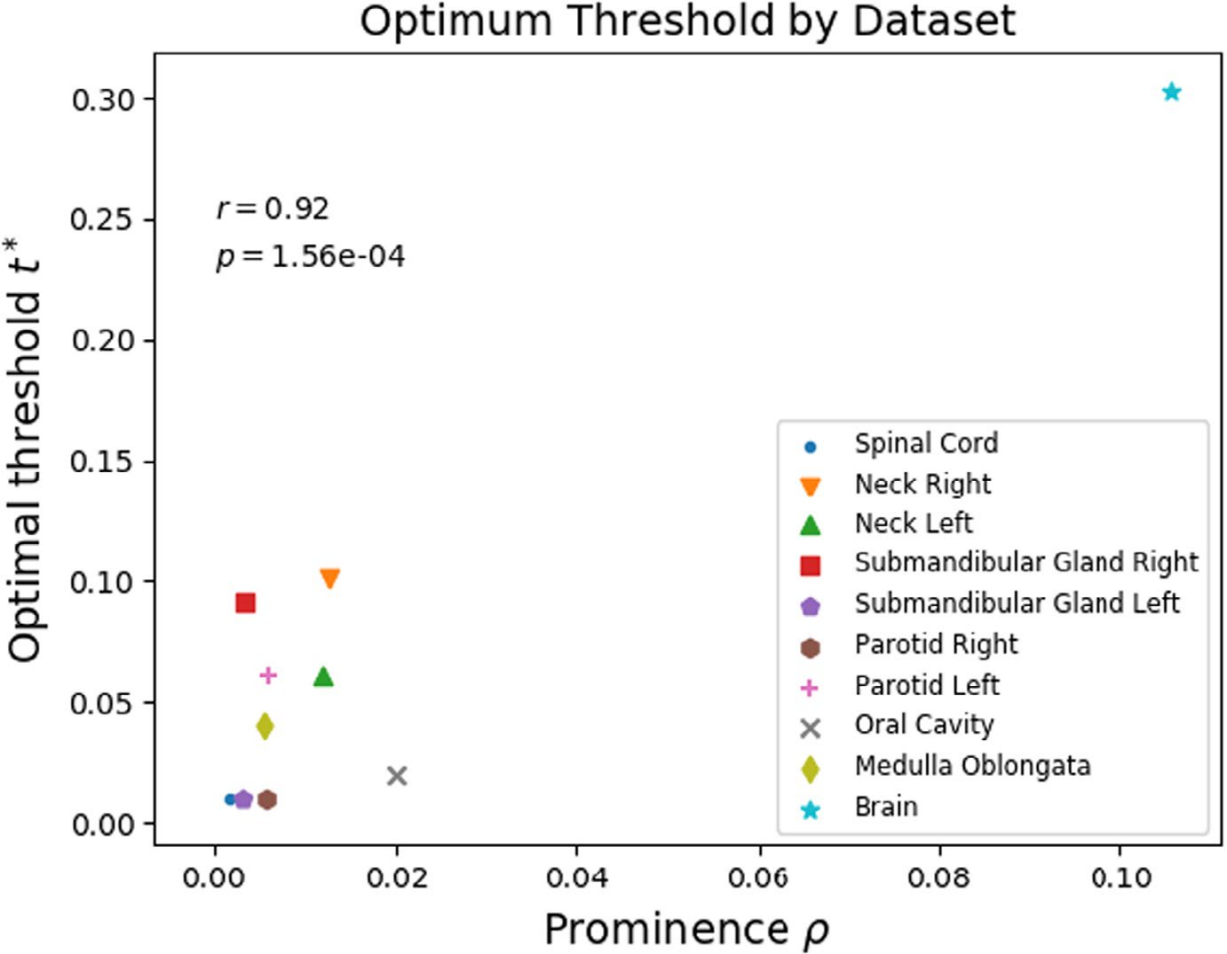
The anatomical segmentation results suggest that the optimum threshold $t^{*}$ and the prominence ρ for our datasets are strongly correlated

The value of the optimal threshold for these datasets appears to represent the model's hesitancy toward calling a pixel “structure” based on the structure's prominence in the training dataset (Figure 16). Based on the anatomical segmentations, there is significant statistical evidence to reject the null hypothesis that prominence ρ and optimal threshold $t^{*}$ are not correlated. There is support for a strong correlation $\frac{dt^{*}}{d\rho }>0$ with Pearson r=0.92 and $p\approx 1.56*10^{‐4}$.

**Figure 17:**
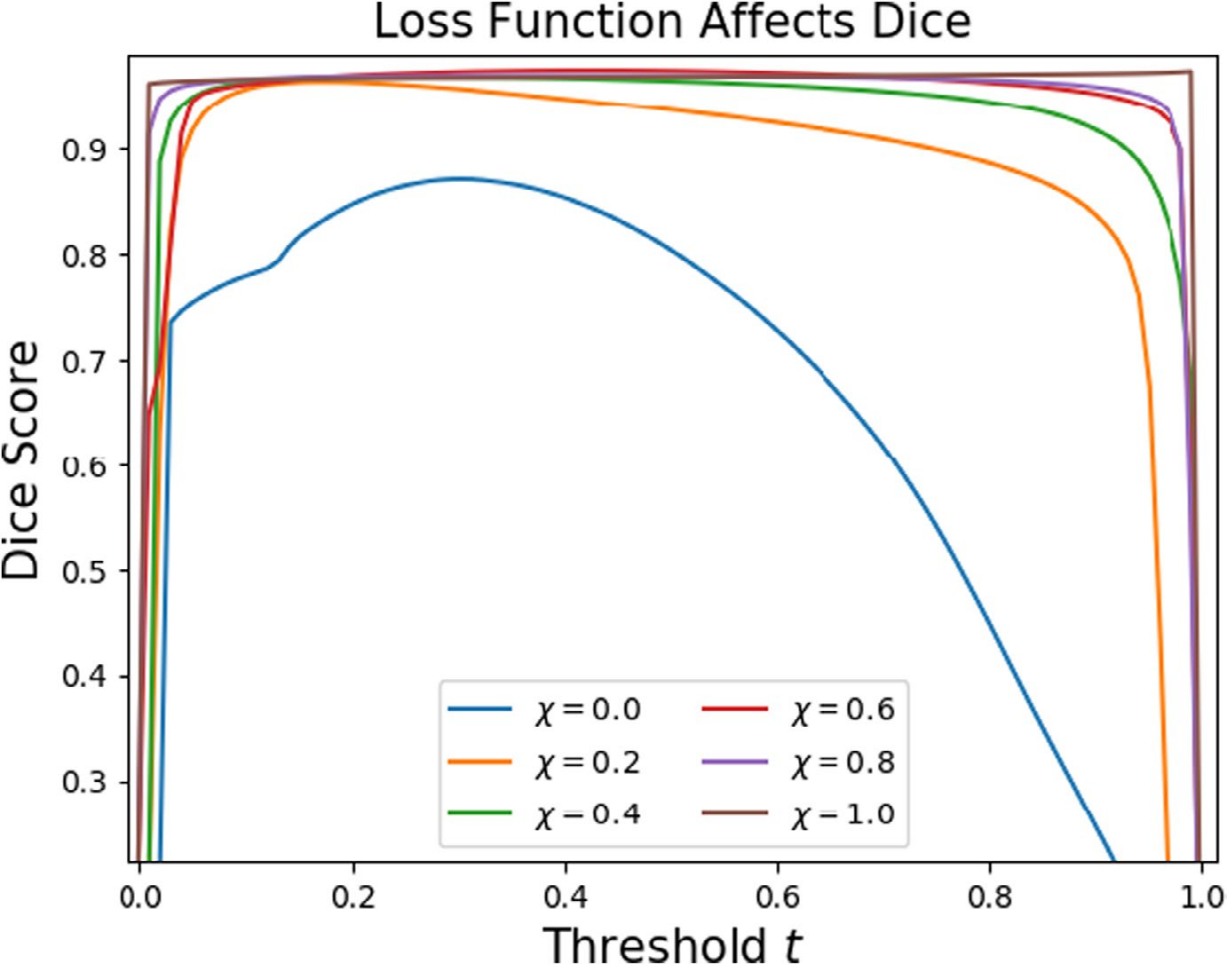
Inclusion of a 2D Dice term in the optimization objective reduces sensitivity to threshold postprocessing. Here, U‐nets trained to segment the brain with a dominant Dice term become insensitive to the threshold hyperparameter with increasing χ

We then observed the stabilizing effect of amending the BCE loss with a 2D Dice term as in Equation (5). As suspected, the sensitivity to threshold postprocessing is significantly reduced with this procedure. The thresholding curves as depicted in Figure 7 are leveled out gradually for variable χ when contouring the brain (Figure 17).

**Figure 18:**
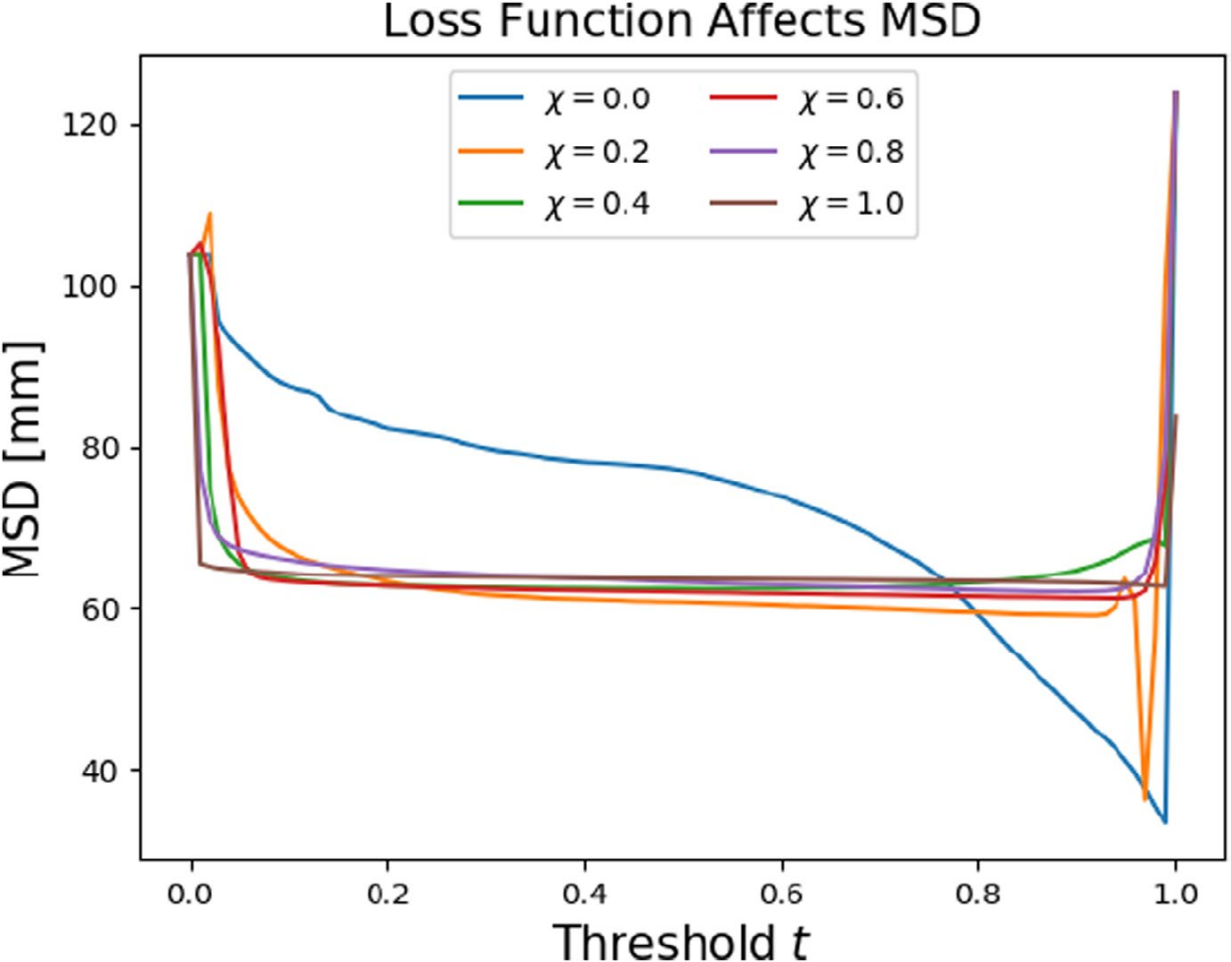
Including a 2D Dice term in the loss function reduces models’ sensitivities to thresholding but does not necessarily improve their overall performance when assessed with different metrics such as MSD

Notice that for an increasing importance of the Dice term (increasing χ), not only is the instability washed out but the overall accuracy of the model at the time of validation is significantly higher. For comparison with a different metric, we perform the same analysis using mean surface distance (Figure 18). We find here that the model trained entirely with a Dice objective was not the best performing model at most thresholds. This is enough to recommend the use of a composite objective function (Equation 5) with a tuned hyperparameter χ. In this case, we found χ=0.2 to be sufficient.

## 4 DISCUSSION

The objective of this work is to bring a common misstep in deep‐learning‐based segmentation to light. We believe that this effort will have a positive impact in the metaheuristics of future segmentation software. We have shown that the relatively simple step of identifying an appropriate threshold can potentially significantly improve segmentation performance.

To the best of our knowledge, this is the first attempt to tangibly show how this specific variety of imbalanced data can affect segmentation accuracy. We show that volume imbalances alone can affect a model's “confidence” and therefore its precision. We do so in the highly clinically relevant case of anatomical CT segmentation, where Dice deviations of only a few percent can have significant implications for the amount of time needed to manually correct automated contours. Although 2D U‐Nets as applied to head and neck segmentation are investigated exclusively in this study, we expect the sensitivities displayed here extend to other sites and architectures. One interesting comparison not included in this work might be between a multilabel segmentation model and a binary classifier with optimized postprocessing. Although binary classification is an easier problem and is typically manageable when there are only a handful of classes (human anatomy), it is susceptible to the postprocessing uncertainties described here such as the choice of a threshold. This is a potential direction of future work and might provide insight for the next generation of commercial contouring software.

One facet of this work that we found particularly insightful was the major discrepancy between model performances as assessed with 3D Dice as compared with MSD and Hausdorff distance (Figures 5, 10, and 11). While the Dice scores would suggest that the brain segmentation model performed better than the spinal cord and submandibular gland models, the MSD scores suggest the exact opposite. For a less‐biased estimate of a model's performance, more than one metric should be considered. This also begs the question of what exactly is desired from clinically implemented segmentation models. Perhaps models optimized with an MSD term in their objective reduce dosimetrists’ time spent touching up contours, or models optimized with a Dice term might be preferred by physicians. A greater understanding of how our metrics and neural network optimization objectives are based in clinical utility is currently needed.

## 5 CONCLUSION

The implementation of intensity‐based postprocessing in deep‐learning‐based biomedical image segmentation was explored. We empirically demonstrate that careless thresholding of deep‐learning‐based segmentation probability maps can have significant implications for the perceived model performance as assessed with the Dice coefficient. Moreover, we provide estimates of typical decreases in validation Dice scores caused by deviations from the optimum value. Specifically, we observe a median Dice decrease of 13.5% when segmenting with a threshold of 0.5 on the held‐out testing datasets. We believe that this is an overestimate of typical error caused by poor thresholding, because head and neck structures are small. We also noticed median Dice decreases of 11.8% with thresholds perturbed only 0.05 from their optimum. The sensitivities of model performance as assessed with 3D Dice were compared with those of MSD and HD. We found in general that these metrics were less affected by suboptimal thresholding, with decreases in accuracy of about 6% compared with 11.8% for the same perturbations about the optima.

A new metric called “prominence” was prescribed to characterize structure volumes in segmentation training datasets. We noticed statistically significant evidence of correlation between the optimal thresholds of our models and the prominence of the Boolean masks used during training. Lastly, we found that inclusion of a 2D Dice term in the optimization objective can significantly reduce sensitivity to thresholding. Despite the desired reduction in sensitivity, we found that the Dice‐optimized models unfavorably prioritize the segmentation of slices with relatively scarce amounts of structure.

Our results suggest that those practicing deep‐learning‐based contouring should consider their postprocessing procedures as a potential avenue for improved performance. For intensity‐based thresholding in biomedical image segmentation, we recommend validating models’ performances with a dense sampling of thresholds between 0 and 1 and performance assessment with a validation dataset. The analyses of model performances with different metrics offer some much‐needed commentary on segmentation performance measures. The compared accuracy of these models when assessed with 3D Dice is sometimes completely different from when assessed with MSD and HD. For a less‐biased understanding of how models are performing, we recommend the use of more than one metric.

## CONFLICT OF INTEREST

None.

## AUTHOR CONTRIBUTIONS

N. B., N. K., R. L., and D. N. devised the project and planned/performed the experiments. T. B. and C. K. collected and preprocessed datasets. P. M., N. P., and M. F. helped interpret results and recommended additional experiments. All authors were involved in contributing to the final manuscript.

## Data Availability

The data that support the findings of this study are available from the corresponding author upon reasonable request.

## Appendix A

The following U‐Net architecture (Table A1) was constructed using PyTorch version 1.8.1. This is a standard model for deep‐learning‐based semantic segmentation popularized by Ronneberger et al. in 2015.

**Table A1:** The model family used in these experiments encodes and decodes image data with 2D convolution and transposed convolution layers. Skip connections provide spatial information from encoding layers to decoding layers of the same resolution

U‐Net Architecture				
Block	Sub‐block	Layer	Output size	Kernels
Encoder block	Input		(1, 128, 128)	
	Convolutional block	(Conv2D, BN, Leaky ReLU, DO)	(16,128,128)	F = 16, K = 3×3, S = 1
	Maxpooling2D		(16,64,64)	K = 2×2, S = 2
	Convolutional block	(Conv2D, BN, Leaky ReLU, DO)	(16,64,64)	F = 16, K = 3×3, S = 1
	Convolutional block	(Conv2D, BN, Leaky ReLU, DO)	(32,64,64)	F = 32, K = 3×3, S = 1
	Maxpooling2D		(32,32,32)	K = 2×2, S = 2
	Convolutional block	(Conv2D, BN, Leaky ReLU, DO)	(32,32,32)	F = 32, K = 3×3, S = 1
	Convolutional block	(Conv2D, BN, Leaky ReLU, DO)	(64,32,32)	F = 64, K = 3×3, S = 1
	Maxpooling2D		(64,16,16)	K = 2×2, S = 2
	Convolutional block	(Conv2D, BN, Leaky ReLU, DO)	(64,16,16)	F = 64, K = 3×3, S = 1
	Convolutional block	(Conv2D, BN, Leaky ReLU, DO)	(128,16,16)	F = 128, K = 3×3, S = 1
	Maxpooling2D		(128,8,8)	K = 2×2, S = 2
	Convolutional block	(Conv2D, BN, Leaky ReLU, DO)	(128,8,8)	F = 128, K = 3×3, S = 1
	Convolutional block	(Conv2D, BN, Leaky ReLU, DO)	(128,8,8)	F = 128, K = 3×3, S = 1
Decoder block	Upsampling block	(ConvTranspose2D, BN, ReLU, DO)	(128,16,16)	F = 128, K = 3×3, S = 2
	Convolutional block*	(Conv2D, BN, Leaky ReLU, DO)	(128,16,16)	F = 128, K = 3×3, S = 1
	Convolutional block	(Conv2D, BN, Leaky ReLU, DO)	(64,16,16)	F = 64, K = 3×3, S = 1
	Upsampling block	(ConvTranspose2D, BN, ReLU, DO)	(64,32,32)	F = 64, K = 3×3, S = 2
	Convolutional block*	(Conv2D, BN, Leaky ReLU, DO)	(64,32,32)	F = 64, K = 3×3, S = 1
	Convolutional block	(Conv2D, BN, Leaky ReLU, DO)	(32,32,32)	F = 32, K = 3×3, S = 1
	Upsampling block	(ConvTranspose2D, BN, ReLU, DO)	(32,64,64)	F = 32, K = 3×3, S = 2
	Convolutional block*	(Conv2D, BN, Leaky ReLU, DO)	(32,64,64)	F = 64, K = 3×3, S = 1
	Convolutional block	(Conv2D, BN, Leaky ReLU, DO)	(16,64,64)	F = 16, K = 3×3, S = 1
	Upsampling block	(ConvTranspose2D, BN, ReLU, DO)	(16,64,64)	F = 16, K = 3×3, S = 2
	Convolutional block	(Conv2D, BN, Leaky ReLU, DO)	(16,128,128)	F = 16, K = 3×3, S = 1
	Output	(Conv2D, Sigmoid)	(1,128,128)	F = 1, K = 3×3, S = 1

BN, batch normalization, DO, dropout.

* Skip connections between Encoding layers of the same image resolution provide half of the features for these layers’ inputs.
